# Antidepressant and antiobesity drug-induced serotonin syndrome complicated by multiple organ injury: A case report

**DOI:** 10.1097/MD.0000000000041626

**Published:** 2025-04-11

**Authors:** Wonkyo Yi, Lae Young Jung, Kyung Pyo Kang, Yisik Kim

**Affiliations:** a Department of Internal Medicine, Research Institute of Clinical Medicine, Jeonbuk National University Medical School, Jeonju, Republic of Korea; b Biomedical Research Institute, Jeonbuk National University Hospital, Jeonju, Republic of Korea.

**Keywords:** case report, multiple organ injury, pulmonary thromboembolism, rhabdomyolysis, serotonin syndrome, stress-induced cardiomyopathy

## Abstract

**Rationale:**

Serotonin syndrome (SS), also referred to as serotonin toxicity, is a potentially life-threatening condition associated with increased serotonergic activity in the central nervous system, but often underdiagnosed due to its variable presentation.

**Patient concerns:**

A 46-year-old woman presented to the emergency department with complaints of fever, confused mental status with slurred speech, agitation. The patient also exhibited lower extremities weakness with spontaneous clonus. The physical examination revealed tachycardia and tachypnea. The patient was taking fluoxetine for depression disorder and anti-obesity medications including topiramate and phentermine. Initial laboratory finding demonstrated severe hypoxemia and rhabdomyolysis with renal dysfunction.

**Diagnoses:**

Spinal tap and brain magnetic resonance imaging showed no clues that could explain the patient’s neuromuscular symptoms. Based on the patient’s overall presentation and use of medications, a diagnosis of SS was made. However, the patient’s inpatient treatment was complicated by pulmonary thromboembolism (PTE) and stress-induced cardiomyopathy (SCM).

**Interventions:**

The patient was intubated and admitted to the intensive care unit for supportive care, including mechanical ventilation and hemodialysis. The patient received an intravenous benzodiazepine and vecuronium for controlling autonomic dysfunction and neuromuscular symptoms. Anticoagulation was also initiated promptly following the diagnosis of PTE.

**Outcomes:**

After intensive supportive care, she was discharged 1 month after admission with normalized left ventricular motion and systolic function.

**Lessons:**

This case highlights that SS should be considered as an etiology of unexplained episodes of altered mental status and neuromuscular abnormalities. Rarely, this condition can be complicated by multiple organ injury, involving conditions such as rhabdomyolysis, renal failure, SCM and PTE. Thus, physicians must be aware of the various and atypical manifestations of serotonin syndrome in order to avoid missing the diagnosis of a potentially fatal condition.

## 1. Introduction

Serotonin syndrome (SS) is a relatively common yet potentially life-threatening condition caused by increased serotonergic activity, usually from serotonergic pharmaceutical agents.^[1]^ The primary features of serotonin syndrome include mental status changes, autonomic hyperactivity, and neuromuscular abnormalities.^[2]^ Drugs associated with SS includes selective serotonin reuptake inhibitors (SSRIs; e.g., fluoxetine), Analgesics (e.g., fentanyl) and numerous other prescription and over-the-counter medications (e.g., amphetamines, ecstasy, opiates, dextromethorphan, etc). The incidence of SS is rising as a consequence of the increased use of antidepressants and anti-obesity medications in clinical practice.

No laboratory tests confirm the diagnosis of the serotonin syndrome. The diagnosis of serotonin syndrome is clinical, which provides challenges in rapid and accurate diagnosis. Healthcare professionals must obtain a detailed medical history and assess the patient’s medication regimen agents because SS can occur from the combination some serotonergic drugs, even when each is used at a therapeutic dose. In our case, a young adult female developed severe serotonin syndrome due to a potential drug interaction between fluoxetine (selective serotonin reuptake inhibitors, SSRI), topiramate (antiepileptic drug with serotonergic properties), and phentermine, an amphetamine-like appetite suppressant with sympathomimetic (substances that mimic adrenaline) properties.^[3–6]^

Treatment for serotonin syndrome involves discontinuing the causative medications and providing supportive care. Severe cases of SS may result in complications, such as multiple organ injury including rhabdomyolysis, renal failure, acute respiratory distress syndrome, coma, and death.^[7]^ Furthermore, the critically ill condition with multiple organ injury can lead to a hypercoagulable condition, which can be associated with pulmonary thromboembolism (PTE).^[8]^ Moreover, increased serotonin activity itself of SS can also potentiate overall procoagulant activity by enhancing the interaction of platelets with tissue factor and platelet activation.^[9]^ In addition to acute physical stress from SS, SSRIs in therapeutic dosages or overdoses have the potential to induce stress-induced cardiomyopathy (SCM).^[10,11]^ The prognosis for serotonin syndrome is favorable if the patient receives adequate treatment. Therefore, physicians must be aware of the various presentations of SS to avoid missing the diagnosis of this life-threatening syndrome.

## 2. Case report

**Figure 1.**
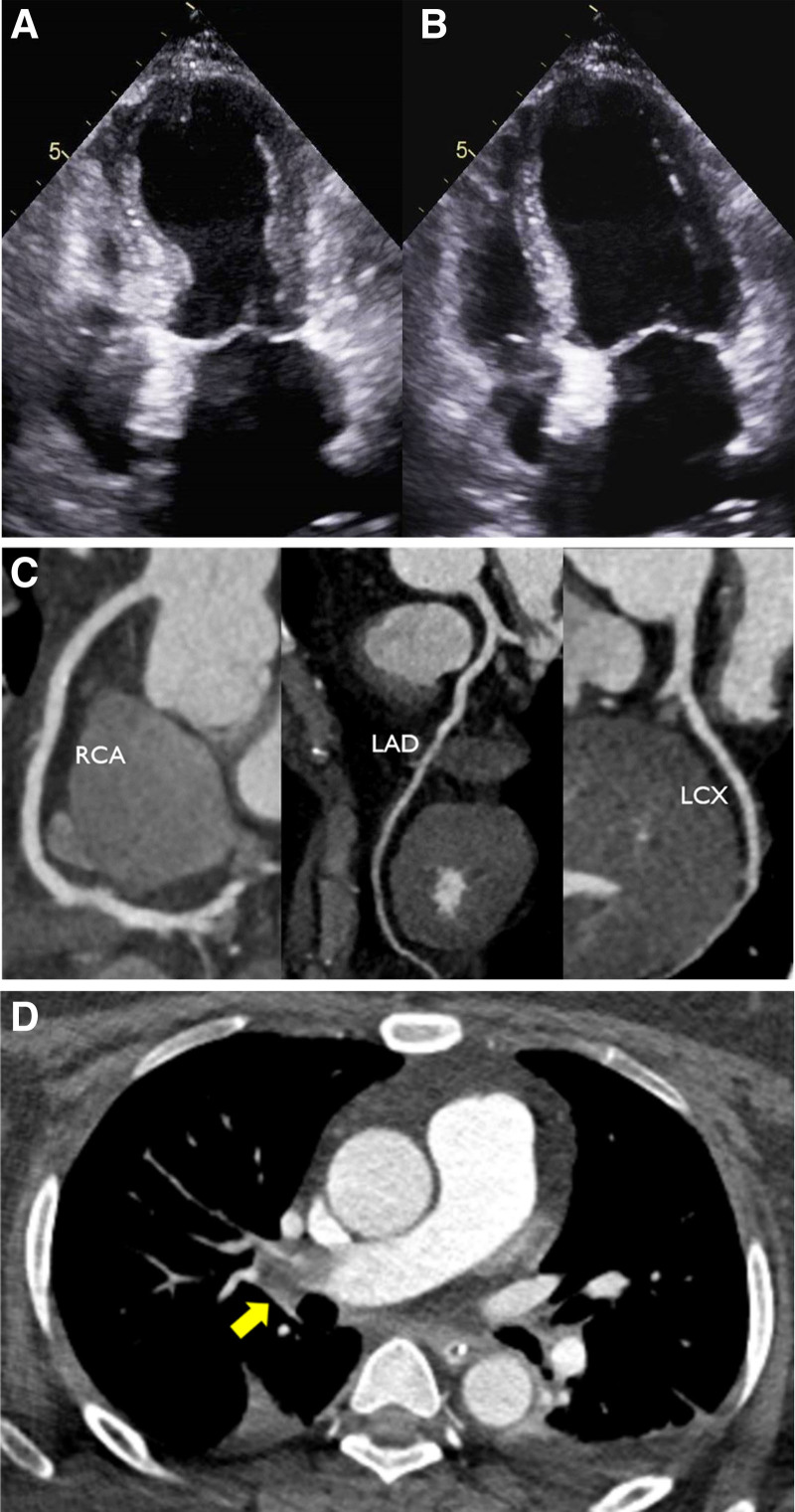
(A, B) The apical-4-chamber view in systole and diastole showing classic apical ballooning with akinesia and hyperdynamic basal segments on echocardiography indicating stress-induced cardiomyopathy. (C) Coronary CT angiography showing normal coronary arteries. (D) Contrast CT showing filling defect (yellow arrow) in right pulmonary artery. CT = computed tomography, RCA = right coronary artery, LAD = left anterior descending coronary artery, LCX = left circumflex artery.

A 46-year-old female with no significant medical history presented with 2-days history of fever and worsening difficulties with speaking and standing. The patient exhibited confused mental status with agitation and the lower extremities weakness with spontaneous clonus. Vital signs revealed that blood pressure of 129/87 mm Hg, heart rate of 140 beats per minute, respiratory rate of 25 breaths per minute, and temperature of 40.0°C. Electrocardiogram showed sinus tachycardia (120–135 bpm). Laboratory findings showed hypokalemia (K 2.2 mmol/L), decreased estimated glomerular filtration rate of 39.3 mL/minute/1.73 m^2^, and marked elevation in liver transaminases (alanine aminotransferase/aspartate aminotransferase of 4153/2872 IU/L), creatinine kinase (5375 U/L), and lactate dehydrogenase (2655 U/L), indicating rhabdomyolysis with acute renal dysfunction (Table 1). Spinal tap and brain magnetic resonance imaging showed no clues that could explain her neuromuscular symptoms. Given those negative workups, a careful review of her medication history revealed that she had been taking fluoxetine (20 mg daily) for depression disorder and recently took anti-obesity medications for 1 month, including phentermine (75 mg daily), topiramate (100 mg daily), and chlorthalidone (12.5 mg daily). The patient’s weight was 80 kg and BMI was 35 kg/m^2^. Based on her overall presentation and use of medications, she was diagnosed with serotonin syndrome (SS). The ineffectiveness of antipyretic agents in reducing fever provided additional evidence for SS. On arrival at the emergency department, her blood oxygen saturation was 77%. However, it rapidly decreased to 50%. The patient was intubated promptly at the emergency department and admitted to the intensive care unit for mechanical ventilation and hemodialysis for acute kidney injury with rhabdomyolysis. Although her neuromuscular symptoms and fever rapidly improved with the intravenous administration of benzodiazepine and vecuronium, tachycardia and tachypnea persisted. Echocardiography revealed akinesia of mid to apical segments with severe left ventricular systolic dysfunction (ejection fraction of 21%; Fig. 1A, B) and coronary computed tomography (CT) angiography showed normal coronary arteries (Fig. 1C), indicating SCM. Echocardiography also revealed right ventricular dysfunction with D-dimer elevated to 20.1 mg/L. Chest CT showed filling defects in the right main pulmonary artery (Fig. 1D). Anticoagulation was initiated for PTE. After intensive supportive care, she was discharged 1 month after admission with normalized left ventricular motion and systolic function. She remained asymptomatic.

**Table 1:** Laboratory results of concern on admission, at discharge.

	Reference	On admission	At discharge
Na (mmol/L)	136–145	136	138
K (mmol/L)	3.5–5.1	2.2	4.6
Cl (mmol/L)	98–107	88	106
P (mg/dL)	2.5–4.5	7.0	4.9
Mg (mg/dL)	1.6–2.4	2.6	1.6
Total protein (g/dL)	6.4–8.3	7.5	6.8
Albumin (g/dL)	3.5–5.2	4.3	3.3
BUN (mg/dL)	8–23	20	6
Creatinine (mg/dL)	0.5–0.9	1.57	0.45
eGFR (mL/min/1.73 m^2^)	60 or higher	39.3	120.5
CRP (mg/L)	0–5.0	25.84	2.94
CK (U/L)	0–170	5375	131
LDH (U/L)	0–250	2655	286
AST (IU/L)	0–32	4153	105
ALT (IU/L)	0–33	2872	24
TSH (mIU/L)	0.27–4.2	1.350	2.270
Free T4 (pmol/L)	12–22	18.30	15.80
Cortisol (mcg/dL)	5–25	20.0	13.5
WBC (×10^3^/µL)	4.8–10.8	9.69	3.01
Hb (g/dL)	12–16	16.1	9.1
Hct (%)	37–48	45.2	29.5
Platelet (×10^3^/µL)	130–450	253	236
PT (s)	9.2–12.6	11.6	N/A
PT INR	0.8–1.1	1.03	N/A
aPTT (s)	24.8–36.1	22.3	N/A
Fibrinogen (mg/dL)	191–471	377	N/A
D-dimer (mg/L FEU)	0–0.49	1.092	N/A
Blood oxygen saturation (%)	95–100	77	100
Ionized calcium (mmol/L)	1.13–1.32	1.04	1.07
PO_2_ (mm Hg)	75–100	56.5	102
Lactate (mmol/L)	0.7–2.5	4.6	1.0
pH	7.35–7.45	7.14	7.4

ALT = alanine transaminase, aPTT = activated partial thromboplastin time, AST = aspartate transaminase, BUN = blood urea nitrogen, CK = creatine kinase, Cl = chloride, CRP = C-reactive protein, eGFR = estimated glomerular filtration rate, FEU = fibrinogen equivalent unit, Hb = hemoglobin, Hct = hematocrit, K = potassium, LDH = lactate dehydrogenase, Mg = magnesium, N/A = not applicable, Na = sodium, P = phosphorus, pH = potential of hydrogen, pO_2_ = partial pressure of oxygen, PT = prothrombin time, PT INR = prothrombin time international normalized ratio, T4 = thyroxine, TSH = thyroid-stimulating hormone, WBC = white blood cells.

## 3. Discussion

Serotonin syndrome (SS) is a potentially life-threatening condition associated with increased serotonergic activity in the peripheral and central nervous system, caused by therapeutic drug use, intentional self-poisoning, or inadvertent interactions between serotonergic drugs.^[7]^ Symptoms can range from mild to fatal and classically include altered mental status, autonomic dysfunction (e.g., hyperthermia, tachycardia, etc), and neuromuscular abnormalities (e.g., clonus, tremor, hyperreflexia, etc).^[2]^ In severe cases, the mortality rate is estimated to reach up to 12%.^[12]^ This case describes a patient who developed severe serotonin syndrome triggered by an interaction between fluoxetine (selective serotonin reuptake inhibitors, SSRI), topiramate (antiepileptic drug with serotonergic properties), and phentermine, an amphetamine-like appetite suppressant with sympathomimetic (substances that mimic adrenaline) properties. Topiramate has been shown to inhibit cytochrome isoform CYP2C19, thereby reducing the clearance of CYP2C19 substrates such as citalopram, amitriptyline, fluoxetine, imipramine, and valproic acid. Consequently, topiramate can potentially induce SS by inhibiting the metabolism of other serotonergic agents.^[3–5]^ Phentermine may also increase 5-HT concentration in the central nervous system. Co-administration of phentermine/topiramate with fluoxetine may potentially induce SS through drug-interaction.^[6]^ Given the continuous discovery of new drug combinations that cause serotonin syndrome and the variability in the occurrence of serotonin syndrome across different drug dosages and combinations, it is imperative that the use of 2 serotonergic drugs be approached with caution.

There is currently no specific antidote for serotonin toxicity. Therefore, it is recommended that early symptom recognition, discontinuing the causative medications and providing supportive care. Serotonin syndrome is usually self-limited and resolves within 24 to 48 hours, but could take 3 to 4 days or longer after the discontinuation of the serotonergic drug.^[13]^ However, as was demonstrated in our case, severe cases of SS may result in complications, such as multiple organ injury including rhabdomyolysis, renal failure, and acute respiratory failure.^[7]^ Although cases of severe rhabdomyolysis as seen in this case is uncommon, rhabdomyolysis associated with severe cases of SS has been reported. Several SSRIs, including sertraline and escitalopram, have been associated with the development of rhabdomyolysis,^[14,15]^ due to possible severe skeletal muscle rigidity leading to muscular necrosis.^[16]^

We diagnosed our patient with SCM based on regional wall motion abnormality (apical ballooning) and normal coronary CT angiography. Although cases complicating by SCM is not common, severe studies have reported SS-associated SCM.^[17,18]^ The hyperadrenergic state caused by serotonin syndrome is consistent with the preceding emotional or physiologic trigger seen in the majority of SCM occurrences.^[19]^ In addition to acute physical stress from SS, SSRIs in therapeutic dosages or overdoses have the potential to induce SCM.^[10,11]^ SS is not only serotonergic but also hyperadrenergic state that may be a trigger of SCM.^[20]^ Additionally, the increased serotonin level resulting from the use of serotonergic agents can directly overstimulate serotonin receptors in the heart, leading to SCM.^[21]^

We now evaluate the available evidence for association of PTE in our case of SS. Firstly, recent reports have indicated that depressive symptoms may increase the risk of stroke by increasing platelet activity due to sympathoadrenal hyperactivity.^[22]^ Depressed patients exhibit greater platelet activation, as demonstrated by increased binding of monoclonal antibody, that is, annexin V protein, in comparison to healthy controls.^[23]^ Platelet 5-hydroxytryptamine-2 receptor binding density, a useful index of serotonin-mediated platelet activation, has been found to be higher in depressed patients than in controls.^[24]^ These findings support the hypothesis that depression increases the risk of hypercoagulability through increased platelet aggregation. Secondly, research has demonstrated that SSRIs do not merely affect neuronal serotonin uptake; they also modulate peripheral serotonin. Serotonin, it itself, is a relatively weak platelet activator. However, when it is present with other proaggregatory factors (e.g., adenosine diphosphate, adrenaline, collagen), serotonin significantly potentiates platelet aggregation.^[25]^ Fluvoxamine, fluoxetine, sertraline, and paroxetine have been shown to reduce platelet and whole blood serotonin concentrations after repeated doses.^[26]^ Antidepressants, particularly SSRIs, have been found to attenuate platelet activation by depleting serotonin storage, which may contribute to a reduction in the risk of hypercoagulability.^[27]^ Several studies have shown that the reduction in platelet and whole blood serotonin concentrations and the inhibitory effect on platelet activation occur after repeated administration of the SSRIs.^[28]^ Furthermore, in our patient, decreased mobility and the critically ill condition with multiple organ injury can be putative risk factors for hypercoagulable condition.^[8]^ To the best of our knowledge, this is the first report of a patient with SS who was complicated by multiple organ injury, including SCM and PTE.

In conclusion, this case highlights that SS should be considered as an etiology of unexplained episodes of altered mental status and neuromuscular abnormalities and the importance of careful use of serotonergic drug combination. Rarely, this condition can be complicated by multiple organ injury, involving conditions such as rhabdomyolysis, renal failure, respiratory failure, SCM and PTE, as seen in our case. Thus, it is crucial for clinicians to be aware of the various and atypical manifestations of serotonin syndrome in order to avoid missing the diagnosis of a potentially fatal condition.

## Author contributions

**Investigation:** Wonkyo Yi, Lae Young Jung, Yisik Kim.

**Conceptualization:** Kyung Pyo Kang, Yisik Kim.

**Data curation:** Lae Young Jung, Kyung Pyo Kang, Yisik Kim.

**Supervision:** Kyung Pyo Kang.

**Writing – original draft:** Wonkyo Yi, Kyung Pyo Kang.

**Writing – review & editing:** Yisik Kim.
